# Monomers for adhesive polymers, 18. Synthesis, photopolymerization and adhesive properties of polymerizable α-phosphonooxy phosphonic acids[Article-fn FN0001]

**DOI:** 10.1080/15685551.2016.1231043

**Published:** 2016-09-29

**Authors:** Yohann Catel, Cédéric Dellsperger, Norbert Moszner

**Affiliations:** ^a^ Ivoclar Vivadent AG, Schaan, Liechtenstein

**Keywords:** Adhesives, dental polymers, photopolymerization, synthesis

## Abstract

Four polymerizable α-phosphonooxy phosphonic acids **7a**, **7b**, **9** and **16** were synthesized in seven steps. They were characterized by ^1^H, ^13^C and ^31^P NMR spectroscopy and by high-resolution mass spectroscopy. The copolymerization of acidic monomers **7a**, **7b**, **9** and **16** with 2-hydroxyethyl methacrylate was studied using a differential scanning calorimeter. Due to the presence of two acidic groups, those monomers are significantly more reactive than 10-methacryloyloxydecylphosphonic acid (MDPA) and 10-methacryloyloxydecyl dihydrogen phosphate (MDP). Self-etch adhesives based on monomers **7a**, **7b**, **9** and **16** were formulated and used to mediate a bond between a dental composite and the dental hard tissues (dentin and enamel). These adhesives exhibit excellent performances and provide significantly higher dentin and enamel shear bond strength than adhesives based on MDP or MDPA.

## 1. Introduction

**Figure 1.**
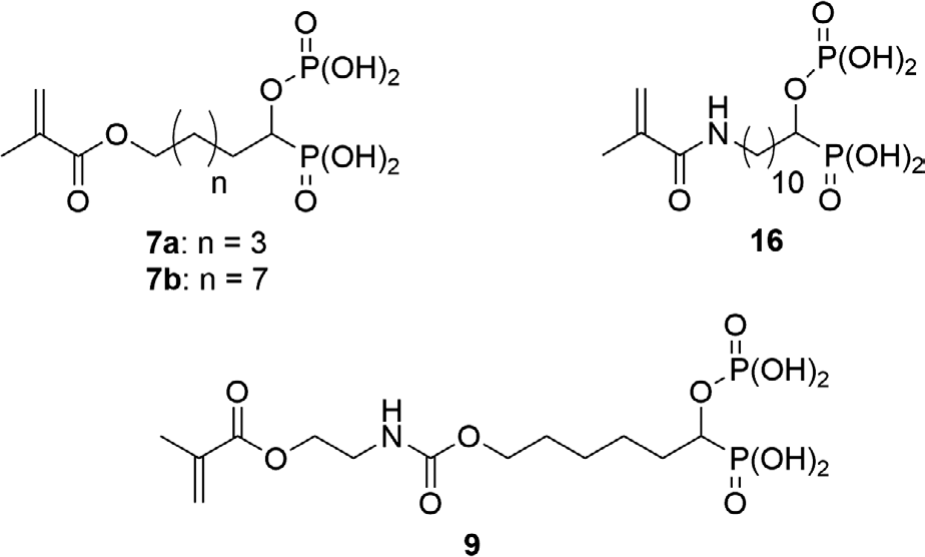
Structure of acidic monomers **7a**, **7b**, **9** and **16**.

Self-etch adhesives (SEAs) are nowadays often used to achieve a bond between the dental hard tissues (dentin and enamel) and a restorative material (composite).[1–3] A SEA is an aqueous formulation containing different monomers (acidic, monofunctional and crosslinking monomers), cosolvents (ethanol, acetone, etc.) and additives (photoinitiator(s), co-initiator(s), stabilizers, fillers, etc.). Contrary to total-etch adhesives, SEAs are able to simultaneously demineralize and infiltrate the dental tissues. The acidic monomer is responsible for the etching of the tooth surface. Monomers such as polymerizable dihydrogen phosphates, carboxylic or phosphonic acids can be found in SEA formulations.[4] In SEAs, the acidic monomer should exhibit good etching properties, sufficient storage stability and a low oral toxicity.[5] It also has to show a high rate of homo- or copolymerization with the comonomers of the formulation. Van Meerbeek et al. [6–9] demonstrated that the ability of the acidic monomer to strongly interact with the calcium of hydroxyapatite (HAP) has a significant impact on the adhesive performance. Therefore, acidic monomers exhibiting strong chelating properties were synthesized and tested in SEAs. It has been demonstrated that the adhesion of SEAs can be improved when polymerizable diphosphonic acids,[10–12] β-ketophosphonic acids [13] or phosphonic acids bearing urea groups [14] are used as acidic monomer. Gem-phosphonate-phosphates were found to be potential antiatherosclerotic agents.[15] Due to the presence of both a phosphonic acid and a dihydrogen phosphate group on the same carbon, such compounds should present excellent chelating properties. To the best of our knowledge, polymerizable gem-phosphonate-phosphates have not yet been reported in the literature. In this context, we took an interest in the synthesis of polymerizable α-phosphonooxy phosphonic acids **7a**, **7b**, **9** and **16** (Figure 1). In this article, the synthesis, photopolymerization behavior and adhesive properties of monomers **7a**, **7b**, **9** and **16** are described.

## 2. Experimental

### 2.1. Abbreviations

Butylated hydroxytoluene (BHT), camphorquinone (CQ), dichloromethane (DCM), double-bond conversion (DBC), N,N′-diethyl-13-bis-(acrylamido)propane (DEBAAP), diethyl ether (Et_2_O), 4-dimethylaminopyridine (DMAP), dimethylformamide (DMF), ethanol (EtOH), ethyl acetate (EA), ethyl 4-(dimethylamino)benzoate (EMBO), geminal (gem), Hydroxyapatite (HAP), 2-hydroxyethyl methacrylate (HEMA), Bis(2,4,6-trimethylbenzoyl)-phenylphosphineoxide (Irgacure 819), bis-(4-methoxybenzoyl)diethylgermanium (Ivocerin^®^), 10-methacryloyloxydecyl dihydrogen phosphate (MDP), 10-methacryloyloxydecylphosphonic acid (MDPA), methanol (MeOH), rate of polymerization (*R*_p_), room temperature (*RT*), shear bond strength (SBS), self-etch adhesive (SEA), tetrabutylammonium fluoride (TBAF), tetrahydrofuran (THF), tetramethylsilane (TMS), trimethylsilyl bromide (TMSBr).

### 2.2. Materials

DCM, THF and MeOH were dried over molecular sieves. TMSBr was distilled prior to use. 2-Isocyanatoethyl methacrylate was purchased from ABCR. *tert*-Butylchlorodiphenylsilane was purchased from TCI. All other reagents were purchased from Sigma–Aldrich and were used without further purification. BHT (Sigma–Aldrich), Bis-GMA (Esschem), CQ (Rahn), DEBAAP (Ivoclar-Vivadent AG), EMBO (Sigma–Aldrich), HEMA (Evonik), Ivocerin^®^ (Ivoclar-Vivadent AG), Irgacure 819 (BASF) and MDP (Orgentis Chemical GmbH) were used in the adhesive formulations and for the photopolymerization studies. MDPA was synthesized according to a procedure reported in the literature.[16] Column chromatography were performed on Macherey-Nagel silica gel 60 (40–63 μm). Thin layer chromatography was performed on silica gel 60 F-254 plates.

### 2.3. Methods

^1^H-NMR, ^13^C-NMR and ^31^P-NMR spectra were recorded on a DPX-400 spectrometer using TMS as internal reference for ^1^H-NMR and ^13^C-NMR chemical shifts and using H_3_PO_4_ (85%) as external reference for ^31^P-NMR chemical shifts. Data are given in the following order: chemical shift in ppm, multiplicity (s, singlet; d, doublet; t, triplet; q, quadruplet; qt, quintuplet; m, multiplet), coupling constant in Hertz, assignment. High-resolution mass spectra (HRMS) were obtained using a Xevo G2-Xs QTof mass spectrometer (Waters).

### 2.4. Syntheses

#### 2.4.1. Synthesis of the acidic monomer **7a**

##### 2.4.1.1. 6-(tert-Butyl-diphenylsilyloxy)hexanoic acid **1a**

*tert*-Butylchlorodiphenylsilane (58.03 g, 0.21 mol) was added, under argon atmosphere, to a solution of 6-hydroxycaproic acid (25.0 g, 0.19 mol) and imidazole (28.98 g, 0.43 mol) in DMF (180 mL). The solution was stirred for 15 h at 50 °C. The solution was poured into 400 mL of brine and the mixture was extracted with EA (3 × 500 mL). The organic layers were gathered and washed with deionized water (2 × 500 mL). The organic layer was dried over sodium sulfate, filtered and concentrated under reduced pressure. The crude product was purified by flash column chromatography (eluent = DCM/MeOH: 98/2). 46.1 g (0.12 mol) of the desired carboxylic acid **1a** were isolated.

Yield: 66%. Aspect: colorless oil. ^1^H-NMR (400 MHz, CDCl_3_): *δ* = 1.04 (s, 9H, CH_3_ *t*Bu); 1.36–1.47 (m, 2H, CH_2_); 1.52–1.67 (m, 4H, CH_2_); 2.33 (t, ^3^ *J*_HH_ = 7.6 Hz, 2H, CH_2_COOH); 3.65 (t, ^3^ *J*_HH_ = 6.4 Hz, 2H, CH_2_OSi); 7.34–7.45 (m, 6H, CHAr); 7.63–7.69 (m, 4H, CHAr).

##### 2.4.1.2. 6-(tert-Butyl-diphenylsilyloxy)hexanoyl chloride **2a**

Oxalyl chloride (2.78 mL, 32.4 mmol) was added dropwise to a solution of carboxylic acid **1a** (10.0 g, 27.0 mmol) in anhydrous toluene (100 mL). The solution was stirred for 4 h at room temperature. The solution was concentrated under reduced pressure. 10.5 g (27.0 mmol) of **2a** were obtained.

Yield: 100%. Aspect: colorless oil. ^1^H NMR (400 MHz, CDCl_3_): *δ* = 1.05 (s, 9H, CH_3_ *t*Bu); 1.37–1.48 (m, 2H, CH_2_); 1.51–1.61 (m, 2H, CH_2_); 1.69 (qt, ^3^ *J*_HH_ = 7.6 Hz, 2H, CH_2_); 2.86 (t, ^3^ *J*_HH_ = 7.4 Hz, 2H, CH_2_COCl); 3.66 (t, ^3^ *J*_HH_ = 6.2 Hz, 2H, CH_2_OSi); 7.35–7.46 (m, 6H, CHAr); 7.62–7.69 (m, 4H, CHAr).

##### 2.4.1.3. Diethyl 6-(tert-butyl-diphenylsilyloxy)-1-oxo-hexylphosphonate **3a**

Triethyl phosphite (4.61 mL, 26.9 mmol) was added dropwise, under argon atmosphere, at 0 °C, to a solution of acyl chloride **2a** (10.5 g, 26.9 mmol) in anhydrous DCM (20 mL). The solution was stirred for 1 h at room temperature. The solution was concentrated under reduced pressure. 13.1 g (26.8 mmol) of α-ketophosphonate **3a** were obtained.

Yield: 100%. Aspect: slightly yellow oil. ^1^H NMR (400 MHz, CDCl_3_): *δ* = 1.04 (s, 9H, CH_3_ *t*Bu); 1.32–1.44 (m, 2H, CH_2_); 1.37 (t, ^3^ *J*_HH_ = 7.1 Hz, 6H, POCH_2_ CH_3_); 1.50–1.67 (m, 4H, CH_2_); 2.83 (t, ^3^ *J*_HH_ = 7.3 Hz, 2H, CH_2_COP); 3.65 (t, ^3^ *J*_HH_ = 6.3 Hz, 2H, CH_2_OSi); 4.17–4.27 (m, 4H, POCH_2_CH_3_); 7.33–7.46 (m, 6H, CHAr); 7.62–7.69 (m, 4H, CHAr). ^31^P NMR (162 MHz, CDCl_3_): *δ* = −2.7. ^13^C NMR (101 MHz, CDCl_3_): *δ* = 16.4 (d, ^3^ *J*_C*p*_ = 5.6 Hz, POCH_2_ CH_3_); 19.2 (SiC(CH_3_)_3_), 22.2 (d, ^3^ *J*_C*p*_ = 3.9 Hz, CH_2_CH_2_COP); 25.2 (CH_2_); 26.9 (SiC(CH_3_)_3_); 32.2 (CH_2_); 43.4 (d, ^2^ *J*_C*p*_ = 54.0 Hz, CH_2_COP); 63.6 (CH_2_OSi); 63.7 (d, ^2^ *J*_C*p*_ = 7.3 Hz, POCH_2_CH_3_); 127.6 (CAr); 129.6 (CAr); 134.0 (CAr); 135.6 (CAr); 211.2 (d, ^1^ *J*_C*p*_ = 165.2 Hz, COP).

##### 2.4.1.4. Diethyl 6-(tert-butyl-diphenylsilyloxy)-1-diethylphosphonooxy-hexylphosphonate **4a**

A solution of α-ketophosphonate **3a** (13.1 g, 26.6 mmol) in Et_2_O (40 mL) was slowly added at 0 °C to a solution of diethyl phosphite (3.43 mL, 26.6 mmol) and diethylamine (2.75 mL, 26.6 mmol) in Et_2_O (60 mL). The reaction mixture was stirred for 30 min at 0 °C and for 24 h at room temperature. The solution was concentrated under reduced pressure. The crude product was purified by flash column chromatography (eluent = EA). 12.0 g (19.1 mmol) of the compound **4a** were isolated.

Yield: 72%. Aspect: slightly yellow oil. ^1^H NMR (400 MHz, CDCl_3_): *δ* = 1.04 (s, 9H, CH_3_ *t*Bu); 1.26–1.66 (m, 18H, CH_2_ and POCH_2_ CH_3_); 1.76–1.99 (m, 2H, CH_2_); 3.65 (t, ^3^ *J*_HH_ = 6.4 Hz, 2H, CH_2_OSi); 4.05–4.25 (m, 8H, POCH_2_CH_3_); 4.56–4.68 (m, 1H, CHP); 7.34–7.45 (m, 6H, CH_Ar_); 7.63–7.69 (m, 4H, CH_Ar_). ^31^P NMR (162 MHz, CDCl_3_): *δ* = −1.0 (d, ^3^ *J*_P*p*_ = 21.6 Hz, CHOP); 20.2 (d, ^3^ *J*_P*p*_ = 21.6 Hz, CHP). ^13^C NMR (101 MHz, CDCl_3_): *δ* = 16.0 (d, ^3^ *J*_C*p*_ = 3.4 Hz, POCH_2_ CH_3_); 16.1 (d, ^3^ *J*_C*p*_ = 3.2 Hz, POCH_2_ CH_3_); 16.4 (d, ^3^ *J*_C*p*_ = 5.6 Hz, POCH_2_ CH_3_); 16.5 (d, ^3^ *J*_C*p*_ = 5.6 Hz, POCH_2_ CH_3_); 19.2 (SiC(CH_3_)_3_), 25.3 (d, ^3^ *J*_C*p*_ = 10.5 Hz, CH_2_CH_2_CHP); 25.6 (CH_2_); 26.9 (SiC(CH_3_)_3_); 31.0 (CH_2_); 32.4 (CH_2_); 62.8 (d, ^2^ *J*_C*p*_ = 6.6 Hz, POCH_2_CH_3_); 63.8 (CH_2_OSi); 64.0 (d, ^2^ *J*_C*p*_ = 5.9 Hz, POCH_2_CH_3_); 73.2 (dd, ^1^ *J*_C*p*_ = 169.5 Hz, ^2^ *J*_C*p*_ = 7.2 Hz, CHP); 127.6 (C_Ar_); 129.5 (C_Ar_); 134.1 (C_Ar_); 135.6 (C_Ar_).

##### 2.4.1.5. Diethyl 6-hydroxy-1-diethylphosphonooxy-hexylphosphonate **5a**

A solution of TBAF (7.23 g, 23.0 mmol) in THF (25 mL) was added dropwise to a solution of compound **4a** (12.0 g, 19.1 mmol) in THF (50 mL). The reaction mixture was stirred for 3 h at room temperature. A saturated solution of ammonium chloride (4 mL) was added. The solution was concentrated under reduced pressure. Deionized water (60 mL) was added and the solution was extracted with EA (3 × 60 mL). The combined organic layers were dried over sodium sulfate and concentrated under reduced pressure. The crude product was purified by flash column chromatography (eluent = EA/MeOH: 8/2). 6.4 g (16.4 mmol) of the desired alcohol **5a** were isolated.

Yield: 86%. Aspect: slightly yellow oil. ^1^H NMR (400 MHz, CDCl_3_): *δ* = 1.26–1.66 (m, 18H, CH_2_ and POCH_2_ CH_3_); 1.83–1.95 (m, 2H); 2.28 (ls, 1H, OH); 3.55–3.66 (m, 2H, CH_2_OH); 4.06–4.22 (m, 8H, POCH_2_CH_3_); 4.57–4.68 (m, 1H, CHP). ^31^P NMR (162 MHz, CDCl_3_): *δ* = −1.0 (d, ^3^ *J*_P*p*_ = 22.1 Hz, CHOP); 20.1 (d, ^3^ *J*_P*p*_ = 22.1 Hz, CHP). ^13^C NMR (101 MHz, CDCl_3_): *δ* = 16.0 (d, ^3^ *J*_C*p*_ = 3.3 Hz, POCH_2_ CH_3_); 16.1 (d, ^3^ *J*_C*p*_ = 3.2 Hz, POCH_2_ CH_3_); 16.4 (d, ^3^ *J*_C*p*_ = 5.6 Hz, POCH_2_ CH_3_); 16.5 (d, ^3^ *J*_C*p*_ = 5.5 Hz, POCH_2_ CH_3_); 24.5 (d, ^3^ *J*_C*p*_ = 10.9 Hz, CH_2_CH_2_CHP); 24.8 (CH_2_); 30.8 (CH_2_); 32.3 (CH_2_); 62.0 (CH_2_OH); 62.8 (d, ^2^ *J*_C*p*_ = 6.2 Hz, POCH_2_CH_3_); 62.9 (d, ^2^ *J*_C*p*_ = 7.1 Hz, POCH_2_CH_3_); 64.1 (d, ^2^ *J*_C*p*_ = 5.7 Hz, POCH_2_CH_3_); 64.1 (d, ^2^ *J*_C*p*_ = 5.7 Hz, POCH_2_CH_3_); 72.7 (dd, ^1^ *J*_C*p*_ = 170.2 Hz, ^2^ *J*_C*p*_ = 7.2 Hz, CHP).

##### 2.4.1.6. Diethyl 6-methacryloyloxy-1-diethylphosphonooxy-hexylphosphonate **6a**

Methacrylic anhydride (1.31 mL, 8.8 mmol) was added, under argon atmosphere, to a solution of alcohol **5a** (3.12 g, 8.0 mmol), DMAP (49 mg, 0.40 mmol) and triethylamine (1.23 mL, 8.8 mmol) in dry DCM (30 mL). The reaction mixture was stirred for 6 h at room temperature. The solution was concentrated under reduced pressure. EA (70 mL) was added and the solution was washed with a saturated solution of sodium bicarbonate (2 × 70 mL). The combined organic layers were dried over sodium sulfate and concentrated under reduced pressure. The crude product was purified by flash column chromatography (eluent = EA/MeOH: 95/5). 2.84 g (6.2 mmol) of monomer **6a** were isolated.

Yield: 78%. Aspect: colorless oil. ^1^H NMR (400 MHz, CDCl_3_): *δ* = 1.28–1.76 (m, 18H, CH_2_ and POCH_2_ CH_3_); 1.81–2.03 (m, 5H, CH_2_ and CH_3_); 4.07–4.28 (m, 10H, POCH_2_CH_3_ and CH_2_OCO); 4.57–4.72 (m, 1H, CHP); 5.53–5.59 (m, 1H, CH_2_=C); 6.10 (ls, 1H, CH_2_=C). ^31^P NMR (162 MHz, CDCl_3_): *δ* = −1.1 (d, ^3^ *J*_P*p*_ = 21.8 Hz, CHOP); 19.9 (d, ^3^ *J*_P*p*_ = 21.8 Hz, CHP). ^13^C NMR (101 MHz, CDCl_3_): *δ* = 16.0 (d, ^3^ *J*_C*p*_ = 4.9 Hz, POCH_2_ CH_3_); 16.1 (d, ^3^ *J*_C*p*_ = 4.8 Hz, POCH_2_ CH_3_); 16.4 (d, ^3^ *J*_C*p*_ = 5.8 Hz, POCH_2_ CH_3_); 16.5 (d, ^3^ *J*_C*p*_ = 5.6 Hz, POCH_2_ CH_3_); 18.3 (CH_3_); 25.0 (d, ^3^ *J*_C*p*_ = 10.5 Hz, CH_2_CH_2_CHP); 25.7 (CH_2_); 28.4 (CH_2_); 30.9 (CH_2_); 62.8 (d, ^2^ *J*_C*p*_ = 6.7 Hz, POCH_2_CH_3_); 64.0 (d, ^2^ *J*_C*p*_ = 5.9 Hz, POCH_2_CH_3_); 64.5 (CH_2_OCO); 73.0 (dd, ^1^ *J*_C*p*_ = 169.9 Hz, ^2^ *J*_C*p*_ = 7.3 Hz, CHP); 125.3 (CH_2_=C); 136.4 (CH_2_=C); 167.5 (C=O).

##### 2.4.1.7. 6-Methacryloyloxy-1-phosphonooxy-hexylphosphonic acid **7a**

TMSBr (4.75 mL, 36.0 mmol) was added, under argon atmosphere, to a solution of monomer **6a** (2.75 g, 6.0 mmol) in anhydrous DCM (40 mL). After stirring for 5 h at 30 °C, the mixture was concentrated under reduced pressure. Methanol (40 mL) was added and the mixture was stirred for 30 min at RT. The solvent was evaporated and the product was dried to a constant weight under vacuum. 1.96 g (5.7 mmol) of the monomer **7a** were isolated.

Yield: 95%. Aspect: highly viscous yellow oil. ^1^H NMR (400 MHz, MeOD): *δ* = 1.28–1.78 (m, 6H, CH_2_); 1.79–1.98 (m, 5H, CH_2_ and CH_3_); 4.15 (t, ^3^ *J*_HH_ = 6.6 Hz, 2H, CH_2_OCO); 4.36–4.48 (m, 1H, CHP); 5.59–5.63 (m, 1H, CH_2_=C); 6.08 (ls, 1H, CH_2_=C). ^31^P NMR (162 MHz, MeOD): *δ* = 0.3 (d, ^3^ *J*_P*p*_ = 18.8 Hz, CHOP); 19.3 (d, ^3^ *J*_P*p*_ = 18.8 Hz, CHP). ^13^C NMR (101 MHz, MeOD): *δ* = 17.0 (CH_3_); 24.7 (d, ^3^ *J*_C*p*_ = 10.3 Hz, CH_2_CH_2_CHP); 25.4 (CH_2_); 28.1 (CH_2_); 30.6 (CH_2_); 64.4 (CH_2_OCO); 73.0 (dd, ^1^ *J*_C*p*_ = 166.1 Hz, ^2^ *J*_C*p*_ = 7.2 Hz, CHP); 124.6 (CH_2_=C); 136.5 (CH_2_=C); 167.5 (C=O). HRMS (*m*/*z*): calcd for C_10_H_19_O_9_P_2_, 345.0504; found, 345.0504 [M-H]^−^.

#### 2.4.2. Synthesis of the acidic monomer **7b**

##### 2.4.2.1. 10-(tert-Butyl-diphenylsilyloxy)decanoic acid **1b**

10-(*tert*-Butyl-diphenylsilyloxy)decanoic acid **1b** was synthesized, from 10-hydroxydecanoic acid (10.0 g, 53.1 mmol), according to the same procedure described for the synthesis of 6-(*tert*-butyl-diphenylsilyloxy)hexanoic acid **1a**. 17.3 g of **1b** were isolated.

Yield: 76%. Aspect: colorless oil. ^1^H NMR (400 MHz, CDCl_3_): *δ* = 1.05 (s, 9H, CH_3_ *t*Bu); 1.20–1.40 (m, 10H, CH_2_); 1.50–1.69 (m, 4H, CH_2_); 2.35 (t, ^3^ *J*_HH_ = 7.6 Hz, 2H, CH_2_COOH); 3.65 (t, ^3^ *J*_HH_ = 6.5 Hz, 2H, CH_2_OSi); 7.35–7.45 (m, 6H, CH_Ar_); 7.65–7.70 (m, 4H, CH_Ar_).

##### 2.4.2.2. 10-(tert-Butyl-diphenylsilyloxy)-decanoyl chloride **2b**

10-(*tert*-Butyl-diphenylsilyloxy)-decanoyl chloride **2b** was synthesized, from 10-(*tert*-butyl-diphenylsilyloxy)decanoic acid **1b** (2.0 g, 4.7 mmol), according to the same procedure described for the synthesis of 6-(*tert*-butyl-diphenylsilyloxy)-hexanoyl chloride **2a**. 2.1 g of **2b** were isolated.

Yield: 100%. Aspect: colorless oil. ^1^H NMR (400 MHz, CDCl_3_): *δ* = 1.06 (s, 9H, CH_3_ *t*Bu); 1.22–1.40 (m, 10H, CH_2_); 1.56 (qt, ^3^ *J*_HH_ = 6.8 Hz, 2H, CH_2_); 1.71 (qt, ^3^ *J*_HH_ = 7.3 Hz, 2H, CH_2_); 2.89 (t, ^3^ *J*_HH_ = 7.3 Hz, 2H, CH_2_COCl); 3.66 (t, ^3^ *J*_HH_ = 6.5 Hz, 2H, CH_2_OSi); 7.35–7.46 (m, 6H, CH_Ar_); 7.65–7.71 (m, 4H, CH_Ar_). ^13^C NMR (101 MHz, CDCl_3_): *δ* = 19.2 (SiC(CH_3_)_3_), 25.1 (CH_2_); 25.7 (CH_2_); 26.9 (CH_3_); 28.4 (CH_2_); 29.0 (CH_2_); 29.2 (CH_2_); 29.3 (CH_2_); 32.5 (CH_2_); 41.1 (CH_2_COCl); 64.0 (CH_2_OSi); 127.6 (C_Ar_); 129.5 (C_Ar_); 134.2 (C_Ar_); 135.6 (C_Ar_); 173.9 (COCl).

##### 2.4.2.3. Diethyl 10-(tert-butyl-diphenylsilyloxy)-1-oxo-decylphosphonate **3b**

Diethyl 10-(*tert*-butyl-diphenylsilyloxy)-1-oxo-decylphosphonate **3b** was synthesized, from 10-(*tert*-butyl-diphenylsilyloxy)-decanoyl chloride **2b** (2.0 g, 4.7 mmol), according to the same procedure described for the synthesis of diethyl 6-(tert-butyl-diphenylsilyloxy)-1-oxo-hexylphosphonate **3a**. 2.4 g of **3b** were isolated.

Yield: 94%. Aspect: colorless oil. ^1^H NMR (400 MHz, CDCl_3_): *δ* = 1.04 (s, 9H, CH_3_ *t*Bu); 1.19–1.38 (m, 10H, CH_2_); 1.37 (t, ^3^ *J*_HH_ = 7.1 Hz, 6H, POCH_2_ CH_3_); 1.49–1.66 (m, 4H, CH_2_); 2.83 (t, ^3^ *J*_HH_ = 7.3 Hz, 2H, CH_2_COP); 3.64 (t, ^3^ *J*_HH_ = 6.5 Hz, 2H, CH_2_OSi); 4.17–4.27 (m, 4H, POCH_2_CH_3_); 7.34–7.45 (m, 6H, CH_Ar_); 7.64–7.69 (m, 4H, CH_Ar_). ^31^P NMR (162 MHz, CDCl_3_): *δ* = −2.7. ^13^C NMR (101 MHz, CDCl_3_): *δ* = 16.4 (d, ^3^ *J*_C*p*_ = 5.6 Hz, POCH_2_ CH_3_); 19.2 (SiC(CH_3_)_3_); 22.4 (d, ^3^ *J*_C*p*_ = 3.8 Hz, CH_2_CH_2_COP); 25.8 (CH_2_); 26.9 (SiC(CH_3_)_3_); 28.9 (CH_2_); 29.2 (CH_2_); 29.3 (CH_2_); 29.4 (CH_2_); 32.5 (CH_2_); 43.4 (d, ^2^ *J*_C*p*_ = 53.8 Hz, CH_2_COP); 63.7 (d, ^2^ *J*_C*p*_ = 7.3 Hz, POCH_2_CH_3_); 64.0 (CH_2_OSi); 127.6 (C_Ar_); 129.5 (C_Ar_); 134.2 (C_Ar_); 135.6 (C_Ar_); 211.4 (d, ^1^ *J*_C*p*_ = 164.8 Hz, COP).

##### 2.4.2.4. Diethyl 10-(tert-butyl-diphenylsilyloxy)-1-diethylphosphonooxy-decylphosphonate **4b**

Diethyl 10-(*tert*-butyl-diphenylsilyloxy)-1-diethylphosphonooxy-decylphosphonate **4b** was synthesized, from diethyl 10-(*tert*-butyl-diphenylsilyloxy)-1-oxo-decylphosphonate **3b** (15.4 g, 28.2 mmol), according to the same procedure described for the synthesis of diethyl 6-(*tert*-butyl-diphenylsilyloxy)-1-diethylphosphonooxy-hexylphosphonate **4a**. 16.1 g of **4b** were isolated.

Eluent: EA. Yield: 83%. Aspect: slightly yellow oil. ^1^H NMR (400 MHz, CDCl_3_): *δ* = 1.04 (s, 9H, CH_3_ *t*Bu); 1.19–1.66 (m, 26H, CH_2_ and POCH_2_ CH_3_); 1.76–1.98 (m, 2H, CH_2_); 3.64 (t, ^3^ *J*_HH_ = 6.5 Hz, 2H, CH_2_OSi); 4.07–4.26 (m, 8H, POCH_2_CH_3_); 4.57–4.69 (m, 1H, CHP); 7.33–7.46 (m, 6H, CH_Ar_); 7.63–7.70 (m, 4H, CH_Ar_). ^31^P NMR (162 MHz, CDCl_3_): *δ* = −1.0 (d, ^3^ *J*_P*p*_ = 21.6 Hz, CHOP); 20.3 (d, ^3^ *J*_P*p*_ = 21.6 Hz, CHP). ^13^C NMR (101 MHz, CDCl_3_): *δ* = 16.0 (d, ^3^ *J*_C*p*_ = 4.4 Hz, POCH_2_ CH_3_); 16.1 (d, ^3^ *J*_C*p*_ = 4.2 Hz, POCH_2_ CH_3_); 16.4 (d, ^3^ *J*_C*p*_ = 5.8 Hz, POCH_2_ CH_3_); 16.5 (d, ^3^ *J*_C*p*_ = 5.7 Hz, POCH_2_ CH_3_); 19.2 (SiC(CH_3_)_3_); 25.4 (d, ^3^ *J*_C*p*_ = 10.7 Hz, CH_2_CH_2_CHP); 25.8 (CH_2_); 26.9 (SiC(CH_3_)_3_); 29.3 (2C, CH_2_); 29.4 (CH_2_); 29.5 (CH_2_); 31.0 (CH_2_); 32.6 (CH_2_); 62.8 (d, ^2^ *J*_C*p*_ = 6.6 Hz, POCH_2_CH_3_); 64.0 (d, ^2^ *J*_C*p*_ = 5.8 Hz, POCH_2_CH_3_); 64.0 (CH_2_OSi); 73.2 (dd, ^1^ *J*_C*p*_ = 169.7 Hz, ^2^ *J*_C*p*_ = 7.4 Hz, CHP); 127.6 (C_Ar_); 129.5 (C_Ar_); 134.2 (C_Ar_); 135.6 (C_Ar_).

##### 2.4.2.5. Diethyl 10-hydroxy-1-diethylphosphonooxy-decylphosphonate **5b**

Diethyl 10-hydroxy-1-diethylphosphonooxy-decylphosphonate 5**b** was synthesized, from diethyl 10-(*tert*-butyl-diphenylsilyloxy)-1-diethylphosphonooxy-decylphosphonate **4b** (3.9 g, 5.7 mmol), according to the same procedure described for the synthesis of diethyl 6-hydroxy-1-diethylphosphonooxy-hexylphosphonate **5a**. 2.35 g of **5b** were isolated.

Eluent: EA/MeOH: 9/1. Yield: 93%. Aspect: colorless oil. ^1^H NMR (400 MHz, CDCl_3_): *δ* = 1.20–1.62 (m, 26H, CH_2_ and POCH_2_ CH_3_); 1.73–1.98 (m, 3H, CH_2_ and OH); 3.59 (t, ^3^ *J*_HH_ = 6.6 Hz, 2H, CH_2_OH); 4.05–4.22 (m, 8H, POCH_2_CH_3_); 4.54–4.66 (m, 1H, CHP). ^31^P NMR (162 MHz, CDCl_3_): *δ* = −1.1 (d, ^3^ *J*_P*p*_ = 21.8 Hz, CHOP); 20.2 (d, ^3^ *J*_P*p*_ = 21.8 Hz, CHP). ^13^C NMR (101 MHz, CDCl_3_): *δ* = 16.0 (d, ^3^ *J*_C*p*_ = 4.5 Hz, POCH_2_ CH_3_); 16.1 (d, ^3^ *J*_C*p*_ = 4.4 Hz, POCH_2_ CH_3_); 16.4 (d, ^3^ *J*_C*p*_ = 5.8 Hz, POCH_2_ CH_3_); 16.5 (d, ^3^ *J*_C*p*_ = 5.7 Hz, POCH_2_ CH_3_); 25.3 (d, ^3^ *J*_C*p*_ = 10.6 Hz, CH_2_CH_2_CHP); 25.7 (CH_2_); 29.1 (CH_2_); 29.2 (CH_2_); 29.3 (CH_2_); 29.4 (CH_2_); 30.9 (CH_2_); 32.7 (CH_2_); 62.8 (d, ^2^ *J*_C*p*_ = 6.2 Hz, POCH_2_CH_3_); 62.9 (CH_2_OH); 64.0 (d, ^2^ *J*_C*p*_ = 6.1 Hz, POCH_2_CH_3_); 73.2 (dd, ^1^ *J*_C*p*_ = 169.8 Hz, ^2^ *J*_C*p*_ = 7.3 Hz, CHP).

##### 2.4.2.6. Diethyl 10-methacryloyloxy-1-diethylphosphonooxy-decylphosphonate **6b**

Diethyl 10-methacryloyloxy-1-diethylphosphonooxy-decylphosphonate **6b** was synthesized, from diethyl 10-hydroxy-1-diethylphosphonooxy-decylphosphonate **5b** (2.3 g, 5.1 mmol), according to the same procedure described for the synthesis of diethyl 6-methacryloyloxy-1-diethylphosphonooxy-hexylphosphonate **6a**. 2.3 g of **5b** were isolated.

Eluent: EA. Yield: 86%. Aspect: colorless oil. ^1^H NMR (400 MHz, CDCl_3_): *δ* = 1.23–1.72 (m, 26H, CH_2_ and POCH_2_ CH_3_); 1.77–1.98 (m, 5H, CH_2_ and CH_3_); 4.08–4.25 (m, 10H, POCH_2_CH_3_ and CH_2_OCO); 4.58–4.68 (m, 1H, CHP); 5.53–5.57 (m, 1H, CH_2_=C); 6.10 (ls, 1H, CH_2_=C). ^31^P NMR (162 MHz, CDCl_3_): *δ* = −1.0 (d, ^3^ *J*_P*p*_ = 21.7 Hz, CHOP); 20.3 (d, ^3^ *J*_P*p*_ = 21.7 Hz, CHP). ^13^C NMR (101 MHz, CDCl_3_): *δ* = 16.0 (d, ^3^ *J*_C*p*_ = 4.4 Hz, POCH_2_ CH_3_); 16.1 (d, ^3^ *J*_C*p*_ = 4.2 Hz, POCH_2_ CH_3_); 16.4 (d, ^3^ *J*_C*p*_ = 5.7 Hz, POCH_2_ CH_3_); 16.5 (d, ^3^ *J*_C*p*_ = 5.8 Hz, POCH_2_ CH_3_); 18.3 (CH_3_); 25.3 (d, ^3^ *J*_C*p*_ = 10.5 Hz, CH_2_CH_2_CHP); 26.0 (CH_2_); 28.6 (CH_2_); 29.2 (2C, CH_2_); 29.3 (CH_2_); 29.4 (CH_2_); 31.0 (CH_2_); 62.8 (d, ^2^ *J*_C*p*_ = 6.6 Hz, POCH_2_CH_3_); 64.0 (d, ^2^ *J*_C*p*_ = 5.9 Hz, POCH_2_CH_3_); 64.8 (CH_2_OCO); 73.2 (dd, ^1^ *J*_C*p*_ = 169.6 Hz, ^2^ *J*_C*p*_ = 7.2 Hz, CHP); 125.2 (CH_2_=C); 136.5 (CH_2_=C); 167.6 (C=O).

##### 2.4.2.7. 10-Methacryloyloxy-1-phosphonooxy-decylphosphonic acid **7b**

10-Methacryloyloxy-1-phosphonooxy-decylphosphonic acid **7b** was synthesized, from diethyl 10-methacryloyloxy-1-diethylphosphonooxy-decylphosphonate **6b** (1.95 g, 3.8 mmol), according to the same procedure described for the synthesis of 6-methacryloyloxy-1-phosphonooxy-hexylphosphonic acid **7a**. 1.52 g of **7b** were isolated.

Yield: 100%. Aspect: highly viscous yellow oil. ^1^H NMR (400 MHz, MeOD): *δ* = 1.26–1.73 (m, 14H, CH_2_); 1.75–1.96 (m, 5H, CH_2_ and CH_3_); 4.13 (t, ^3^ *J*_HH_ = 6.6 Hz, 2H, CH_2_OCO); 4.35–4.46 (m, 1H, CHP); 5.59–5.62 (m, 1H, CH_2_=C); 6.07 (ls, 1H, CH_2_=C). ^31^P NMR (162 MHz, MeOD): *δ* = 0.3 (d, ^3^ *J*_P*p*_ = 18.7 Hz, CHOP); 19.5 (d, ^3^ *J*_P*p*_ = 18.7 Hz, CHP). ^13^C NMR (101 MHz, MeOD): *δ* = 17.1 (CH_3_); 25.1 (d, ^3^ *J*_C*p*_ = 10.4 Hz, CH_2_CH_2_CHP); 25.7 (CH_2_); 28.3 (CH_2_); 28.9 (CH_2_); 29.0 (CH_2_); 29.1 (CH_2_); 29.2 (CH_2_); 30.7 (CH_2_); 64.6 (CH_2_OCO); 73.2 (dd, ^1^ *J*_C*p*_ = 165.9 Hz, ^2^ *J*_C*p*_ = 7.2 Hz, CHP); 124.7 (CH_2_=C); 136.5 (CH_2_=C); 167.5 (C=O). HRMS (*m*/*z*): calcd for C_14_H_27_O_9_P_2_, 401.1130; found, 401.1127 [M-H]^−^.

#### 2.4.3. Synthesis of the acidic monomer **9**

##### 2.4.3.1. Diethyl 6-[2-(methacryloyloxyethylamino)carbonyloxy]-1-diethylphosphonooxy-hexylphosphonate **8**

A solution of dibutyltin dilaurate (25.5 mg, 0.041 mmol) in anhydrous DCM (2.0 mL) was added, under argon atmosphere, to a solution of diethyl 6-hydroxy-1-diethylphosphonooxy-hexylphosphonate **5a** (3.15 g, 8.1 mmol) in anhydrous DCM (10.0 mL). 2-Isocyanatoethyl methacrylate (1.14 mL, 8.1 mmol) was subsequently added dropwise to the mixture. The solution was stirred for 3 h at RT and concentrated under reduced pressure. The crude product was purified by flash column chromatography (eluent: EA/MeOH: 9/1). 4.3 g of the desired compound were isolated.

Yield: 93%. Aspect: slightly yellow oil. ^1^H NMR (400 MHz, CDCl_3_): *δ* = 1.28–1.74 (m, 18H, CH_2_ and POCH_2_ CH_3_); 1.80–1.98 (m, 5H, CH_2_ and CH_3_); 3.40–3.54 (m, 2H, CH_2_NH); 4.00–4.26 (m, 12H, POCH_2_CH_3_, CH_2_OCONH and CH_2_OCO); 4.56–4.67 (m, 1H, CHP); 5.24 (s, 1H, NH); 5.57–5.61 (m, 1H, CH_2_=C); 6.13 (ls, 1H, CH_2_=C). ^31^P NMR (162 MHz, CDCl_3_): *δ* = −1.1 (d, ^3^ *J*_P*p*_ = 22.2 Hz, CHOP); 20.1 (d, ^3^ *J*_P*p*_ = 22.2 Hz, CHP). ^13^C NMR (101 MHz, CDCl_3_): *δ* = 16.0 (d, ^3^ *J*_C*p*_ = 4.6 Hz, POCH_2_ CH_3_); 16.1 (d, ^3^ *J*_C*p*_ = 4.4 Hz, POCH_2_ CH_3_); 16.4 (d, ^3^ *J*_C*p*_ = 5.8 Hz, POCH_2_ CH_3_); 16.5 (d, ^3^ *J*_C*p*_ = 5.8 Hz, POCH_2_ CH_3_); 18.3 (CH_3_); 25.0 (d, ^3^ *J*_C*p*_ = 10.5 Hz, CH_2_CH_2_CHP); 25.7 (CH_2_); 28.5 (CH_2_); 30.8 (CH_2_); 40.0 (CH_2_NH); 62.8 (d, ^2^ *J*_C*p*_ = 6.1 Hz, POCH_2_CH_3_); 62.8 (d, ^2^ *J*_C*p*_ = 7.2 Hz, POCH_2_CH_3_); 63.8 (CH_2_OCO); 64.1 (d, ^2^ *J*_C*p*_ = 6.1 Hz, POCH_2_CH_3_); 65.0 (CH_2_OCO); 73.0 (dd, ^1^ *J*_C*p*_ = 169.9 Hz, ^2^ *J*_C*p*_ = 7.3 Hz, CHP); 126.0 (CH_2_=C); 136.0 (CH_2_=C); 156.7 (C=O); 167.2 (C=O).

##### 2.4.3.2. 6-[2-(Methacryloyloxyethylamino)carbonyloxy]-1-phosphonooxy-hexylphosphonic acid **9**

6-[(2-Methacryloyloxyethylamino)-carbonyloxy]-1-phosphonooxy-hexylphosphonic acid **9** was synthesized, from diethyl 6-[(2-methacryloyloxyethylamino)-carbonyloxy]-1-diethylphosphonooxy-hexylphosphonate **8** (1.0 g, 1.8 mmol), according to the same procedure described for the synthesis of 6-methacryloyloxy-1-phosphonooxy-hexylphosphonic acid **7a**. 750 mg of **9** were isolated.

Yield: 94%. Aspect: highly viscous yellow oil. ^1^H NMR (400 MHz, MeOD): *δ* = 1.26–1.74 (m, 6H, CH_2_); 1.77–1.97 (m, 5H, CH_2_ and CH_3_); 3.38 (t, ^3^ *J*_HH_ = 5.5 Hz, 2H, CH_2_NH); 4.03 (t, ^3^ *J*_HH_ = 6.5 Hz, 2H, CH_2_OCO); 4.17 (t, ^3^ *J*_HH_ = 5.5 Hz, 2H, CH_2_OCONH); 4.34–4.47 (m, 1H, CHP); 5.61–5.65 (m, 1H, CH_2_=C); 6.11 (ls, 1H, CH_2_=C). ^31^P NMR (162 MHz, MeOD): *δ* = 0.3 (CHOP); 19.3 (CHP). ^13^C NMR (101 MHz, MeOD): *δ* = 17.0 (CH_3_); 24.8 (d, ^3^ *J*_C*p*_ = 10.4 Hz, CH_2_CH_2_CHP); 25.3 (CH_2_); 28.6 (CH_2_); 30.6 (CH_2_); 39.3 (CH_2_NH); 63.2 (CH_2_OCO); 64.5 (CH_2_OCO); 73.0 (dd, ^1^ *J*_C*p*_ = 166.0 Hz, ^2^ *J*_C*p*_ = 7.2 Hz, CHP); 125.1 (CH_2_=C); 136.2 (CH_2_=C); 157.9 (C=O); 167.3 (C=O). HRMS (*m*/*z*): calcd for C_13_H_24_NO_11_P_2_, 432.0825; found, 432.0822 [M-H]^−^.

#### 2.4.4. Synthesis of the acidic monomer **16**

##### 2.4.4.1. 11-Phtalimido-undecanoic acid **10**

A mixture of 11-aminoundecanoic acid (3.04 g, 15.1 mmol) and phthalic anhydride (2.24 g, 15.1 mmol) was heated for 1 h at 150 °C. DCM (25 mL) was added and the resulting solution was dried over sodium sulfate. The solution was filtered and concentrated under reduced pressure. 4.9 g (14.8 mmol) of carboxylic acid **10** were obtained.

Yield: 98%. Aspect: white solid. ^1^H NMR (400 MHz, CDCl_3_): *δ* = 1.22–1.40 (m, 12H, CH_2_); 1.56–1.73 (m, 4H, CH_2_); 2.34 (t, ^3^ *J*_HH_ = 7.5 Hz, 2H, CH_2_COOH); 3.67 (t, ^3^ *J*_HH_ = 7.3 Hz, 2H, CH_2_ N); 7.67–7.74 (m, 2H, CHAr); 7.81–7.87 (m, 2H, CHAr). ^13^C NMR (101 MHz, CDCl_3_): *δ* = 24.7 (CH_2_); 26.8 (CH_2_); 28.6 (CH_2_); 29.0 (CH_2_); 29.1 (CH_2_); 29.2 (CH_2_); 29.3 (CH_2_); 29.4 (CH_2_); 34.0 (CH_2_COOH); 38.1 (CH_2_ N); 123.2 (CAr); 132.2 (CAr); 133.8 (CAr); 168.5 (C=O); 179.7 (C=O).

##### 2.4.4.2. 11-Phtalimido-undecanoyl chloride **11**

11-Phtalimido-undecanoyl chloride **11** was synthesized, from 11-phtalimido-undecanoic acid **10** (8.4 g, 25.3 mmol), according to the same procedure described for the synthesis of 6-(*tert*-butyl-diphenylsilyloxy)hexanoyl chloride **2a**. 8.9 g of **11** were isolated.

Yield: 100%. Aspect: colorless oil. ^1^H NMR (400 MHz, CDCl_3_): *δ* = 1.22–1.39 (m, 12H, CH_2_); 1.58–1.77 (m, 4H, CH_2_); 2.87 (t, ^3^ *J*_HH_ = 7.3 Hz, 2H, CH_2_COCl); 3.67 (t, ^3^ *J*_HH_ = 7.3 Hz, 2H, CH_2_ N); 7.68–7.74 (m, 2H, CHAr); 7.81–7.88 (m, 2H, CHAr).

##### 2.4.4.3. Diethyl 11-phtalimido-1-oxo-undecylphosphonate **12**

Diethyl 11-phtalimido-1-oxo-undecylphosphonate **12** was synthesized, from 11-phtalimido-undecanoyl chloride **11** (1.00 g, 2.9 mmol), according to the same procedure described for the synthesis of diethyl 6-(*tert*-butyl-diphenylsilyloxy)-1-oxo-hexylphosphonate **3a**. 1.22 g of **12** were isolated.

Yield: 95%. Aspect: slightly yellow oil. ^1^H NMR (400 MHz, CDCl_3_): *δ* = 1.20–1.35 (m, 12H, CH_2_); 1.36 (t, ^3^ *J*_HH_ = 7.1 Hz, 6H, POCH_2_ CH_3_); 1.53–1.72 (m, 4H, CH_2_); 2.80 (t, ^3^ *J*_HH_ = 7.3 Hz, 2H, CH_2_COP); 3.66 (t, ^3^ *J*_HH_ = 7.3 Hz, 2H, CH_2_ N); 4.14–4.26 (m, 4H, POCH_2_CH_3_); 7.66–7.72 (m, 2H, CH_Ar_); 7.79–7.85 (m, 2H, CH_Ar_). ^31^P NMR (162 MHz, CDCl_3_): *δ* = −2.7. ^13^C NMR (101 MHz, CDCl_3_): *δ* = 16.4 (d, ^3^ *J*_C*p*_ = 5.6 Hz, POCH_2_ CH_3_); 22.4 (d, ^3^ *J*_C*p*_ = 3.7 Hz, CH_2_CH_2_COP); 26.8 (CH_2_); 28.6 (CH_2_); 28.9 (CH_2_); 29.1 (CH_2_); 29.2 (CH_2_); 29.3 (CH_2_); 29.4 (CH_2_); 38.0 (CH_2_ N); 43.3 (d, ^2^ *J*_C*p*_ = 54.0 Hz, CH_2_COP); 63.7 (d, ^2^ *J*_C*p*_ = 7.3 Hz, POCH_2_CH_3_); 123.1 (C_Ar_); 132.2 (C_Ar_); 133.8 (C_Ar_); 168.5 (C_Ar_); 211.3 (d, ^1^ *J*_C*p*_ = 165.0 Hz, COP).

##### 2.4.4.4. Diethyl 11-phtalimido-1-diethylphosphonooxy-undecylphosphonate **13**

Diethyl 11-phtalimido-1-diethylphosphonooxy-undecylphosphonate **13** was synthesized, from diethyl 11-phtalimido-1-oxo-undecylphosphonate **12** (11.5 g, 25.5 mmol), according to the same procedure described for the synthesis of diethyl 6-(*tert*-butyl-diphenylsilyloxy)-1-diethylphosphonooxy-hexylphosphonate **4a**. 10.5 g of **13** were isolated.

Eluent: EA. Yield: 70%. Aspect: slightly yellow oil. ^1^H NMR (400 MHz, CDCl_3_): *δ* = 1.18–1.74 (m, 28H, POCH_2_ CH_3_ and CH_2_); 1.76–1.98 (m, 2H, CH_2_); 3.67 (t, ^3^ *J*_HH_ = 7.2 Hz, 2H, CH_2_ N); 4.07–4.26 (m, 8H, POCH_2_CH_3_); 4.57–4.68 (m, 1H, CHP); 7.68–7.73 (m, 2H, CH_Ar_); 7.81–7.87 (m, 2H, CH_Ar_). ^31^P NMR (162 MHz, CDCl_3_): *δ* = −1.0 (d, ^3^ *J*_P*p*_ = 21.6 Hz, CHOP); 20.3 (d, ^3^ *J*_P*p*_ = 21.6 Hz, CHP). ^13^C NMR (101 MHz, CDCl_3_): *δ* = 16.0 (d, ^3^ *J*_C*p*_ = 4.9 Hz, POCH_2_ CH_3_); 16.1 (d, ^3^ *J*_C*p*_ = 4.7 Hz, POCH_2_ CH_3_); 16.4 (d, ^3^ *J*_C*p*_ = 5.8 Hz, POCH_2_ CH_3_); 16.5 (d, ^3^ *J*_C*p*_ = 5.5 Hz, POCH_2_ CH_3_); 25.4 (d, ^3^ *J*_C*p*_ = 10.4 Hz, CH_2_CH_2_CHP); 26.9 (CH_2_); 28.6 (CH_2_); 29.1 (CH_2_); 29.2 (CH_2_); 29.3 (CH_2_); 29.4 (CH_2_); 31.1 (CH_2_); 38.0 (CH_2_ N); 62.7 (d, ^2^ *J*_C*p*_ = 6.7 Hz, POCH_2_CH_3_); 63.9 (d, ^2^ *J*_C*p*_ = 5.9 Hz, POCH_2_CH_3_); 73.2 (dd, ^1^ *J*_C*p*_ = 169.7 Hz, ^2^ *J*_C*p*_ = 7.4 Hz, CHP); 123.1 (C_Ar_); 132.2 (C_Ar_); 133.8 (C_Ar_); 168.4 (C_Ar_).

##### 2.4.4.5. Diethyl 11-amino-1-diethylphosphonooxy-undecylphosphonate **14**

Hydrazine monohydrate (0.13 mL, 2.54 mmol) was added to a solution of compound **13** (1.00 g, 1.70 mmol) in EtOH (10 mL). The mixture was refluxed for 2 h and the solvent removed under reduced pressure. A solution of NaOH (2 N in distilled water, 20 mL) was added and the mixture was extracted with Et_2_O (3 × 15 mL). The combined organic layers were dried over sodium sulfate and the solvent was removed under reduced pressure. 650 mg (1.41 mmol) of the desired product was isolated.

Yield: 83%. Aspect: slightly yellow oil. ^1^H NMR (400 MHz, CDCl_3_): *δ* = 1.20–1.66 (m, 30H, POCH_2_ CH_3_, CH_2_ and NH_2_); 1.77–1.99 (m, 2H, CH_2_); 2.67 (t, ^3^ *J*_HH_ = 6.9 Hz, 2H, CH_2_NH_2_); 4.07–4.27 (m, 8H, POCH_2_CH_3_); 4.56–4.69 (m, 1H, CHP). ^31^P NMR (162 MHz, CDCl_3_): *δ* = −1.0 (d, ^3^ *J*_P*p*_ = 21.8 Hz, CHOP); 20.3 (d, ^3^ *J*_P*p*_ = 21.8 Hz, CHP). ^13^C NMR (101 MHz, CDCl_3_): *δ* = 16.0 (d, ^3^ *J*_C*p*_ = 4.5 Hz, POCH_2_ CH_3_); 16.1 (d, ^3^ *J*_C*p*_ = 4.4 Hz, POCH_2_ CH_3_); 16.4 (d, ^3^ *J*_C*p*_ = 5.7 Hz, POCH_2_ CH_3_); 16.5 (d, ^3^ *J*_C*p*_ = 5.5 Hz, POCH_2_ CH_3_); 25.3 (d, ^3^ *J*_C*p*_ = 10.6 Hz, CH_2_CH_2_CHP); 26.9 (CH_2_); 29.2 (CH_2_); 29.3 (CH_2_); 29.4 (CH_2_); 29.5 (CH_2_); 31.0 (CH_2_); 33.8 (CH_2_); 40.3 (CH_2_ N); 62.7 (d, ^2^ *J*_C*p*_ = 6.3 Hz, POCH_2_CH_3_); 63.9 (d, ^2^ *J*_C*p*_ = 5.9 Hz, POCH_2_CH_3_); 73.2 (dd, ^1^ *J*_C*p*_ = 169.4 Hz, ^2^ *J*_C*p*_ = 7.3 Hz, CHP).

##### 2.4.4.6. Diethyl 11-methacrylamido-1-diethylphosphonooxy-undecylphosphonate **15**

Methacryloyl chloride (0.23 mL, 2.29 mmol) was added, under argon atmosphere and at 0 °C, to a solution of amine **14** (1.00 g, 2.18 mmol) and triethylamine (0.33 mL, 2.39 mmol) in dry DCM (9 mL). The reaction mixture was stirred for 6 h at RT. The solution was washed with HCl 1 N (10 mL), NaOH 1 N (10 mL) and brine (10 mL). The organic layer was dried over sodium sulfate and concentrated under reduced pressure. The crude product was purified by flash column chromatography (eluent = EA/MeOH: 95/5). 920 mg (1.74 mmol) of monomer **15** were isolated.

Yield: 80%. Aspect: slightly yellow oil. ^1^H NMR (400 MHz, CDCl_3_): *δ* = 1.19–1.64 (m, 28H, POCH_2_ CH_3_ and CH_2_); 1.75–1.94 (m, 2H, CH_2_); 1.94 (s, 3H, CH_3_); 3.28 (q, ^3^ *J*_HH_ = 6.7 Hz, 2H, CH_2_ N); 4.05–4.23 (m, 8H, POCH_2_CH_3_); 4.55–4.66 (m, 1H, CHP); 5.28 (s, 1H, C=CH_2_); 5.64 (s, 1H, C=CH_2_); 5.85 (ls, 1H, NH). ^31^P NMR (162 MHz, CDCl_3_): *δ* = −1.1 (d, ^3^ *J*_P*p*_ = 21.8 Hz, CHOP); 20.2 (d, ^3^ *J*_P*p*_ = 21.8 Hz, CHP). ^13^C NMR (101 MHz, CDCl_3_): *δ* = 16.0 (d, ^3^ *J*_C*p*_ = 4.5 Hz, POCH_2_ CH_3_); 16.1 (d, ^3^ *J*_C*p*_ = 4.4 Hz, POCH_2_ CH_3_); 16.4 (d, ^3^ *J*_C*p*_ = 5.8 Hz, POCH_2_ CH_3_); 16.5 (d, ^3^ *J*_C*p*_ = 5.7 Hz, POCH_2_ CH_3_); 18.7 (CH_3_); 25.3 (d, ^3^ *J*_C*p*_ = 10.5 Hz, CH_2_CH_2_CHP); 26.9 (CH_2_); 29.1 (CH_2_); 29.2 (CH_2_); 29.3 (CH_2_); 29.4 (CH_2_); 29.4 (CH_2_); 29.5 (CH_2_); 31.0 (CH_2_); 39.7 (CH_2_ N); 62.8 (d, ^2^ *J*_C*p*_ = 6.2 Hz, POCH_2_CH_3_); 62.9 (d, ^2^ *J*_C*p*_ = 7.3 Hz, POCH_2_CH_3_); 64.0 (d, ^2^ *J*_C*p*_ = 5.9 Hz, POCH_2_CH_3_); 73.2 (dd, ^1^ *J*_C*p*_ = 169.5 Hz, ^2^ *J*_C*p*_ = 7.2 Hz, CHP); 119.0 (C=CH_2_); 140.3 (C=CH_2_); 168.4 (C=O).

##### 2.4.4.7. 11-Methacrylamido-1-phosphonooxy-undecylphosphonic acid **16**

11-Methacrylamido-1-phosphonooxy-undecylphosphonic acid **16** was synthesized, from diethyl 11-methacrylamido-1-diethylphosphonooxy-undecylphosphonate **15** (900 mg, 1.71 mmol), according to the same procedure described for the synthesis of 6-methacryloyloxy-1-phosphonooxy-hexylphosphonic acid **7a**. 710 mg of **16** were isolated.

Yield: 100%. Aspect: highly viscous slightly yellow oil. ^1^H NMR (400 MHz, MeOD): *δ* = 1.15–1.59 (m, 16H, CH_2_); 1.65–1.87 (m, 5H, CH_2_ and CH_3_); 3.12 (t, ^3^ *J*_HH_ = 7.2 Hz, 2H, CH_2_ N); 4.25–4.35 (m, 1H, CHP); 5.25 (s, 1H, C=CH_2_); 5.56 (s, 1H, C=CH_2_). ^31^P NMR (162 MHz, MeOD): *δ* = 0.3 (d, ^3^ *J*_P*p*_ = 18.6 Hz, CHOP); 19.5 (d, ^3^ *J*_P*p*_ = 18.6 Hz, CHP). ^13^C NMR (101 MHz, MeOD): *δ* = 15.8 (CH_3_); 23.5 (d, ^3^ *J*_C*p*_ = 10.3 Hz, CH_2_CH_2_CHP); 25.0 (CH_2_); 27.3 (CH_2_); 27.4 (CH_2_); 27.5 (CH_2_); 27.6 (CH_2_); 27.6 (CH_2_); 29.1 (CH_2_); 37.8 (CH_2_ N); 71.6 (dd, ^1^ *J*_C*p*_ = 165.9 Hz, ^2^ *J*_C*p*_ = 7.2 Hz, CHP); 117.4 (C=CH_2_); 138.3 (C=CH_2_); 168.4 (C=O). HRMS (*m*/*z*): calcd for C_15_H_30_NO_8_P_2_, 414.1447; found, 414.1443 [M-H]^−^.

### 2.5. Photopolymerization procedure

Photopolymerizations were carried out on a Perkin Elmer differential scanning calorimeter (DSC), Pyris Diamond. 0.5 mol% of Ivocerin^®^ (photoinitiator) were added to each comonomer mixture. A sample (ca. 0.8 mg) of each mixture was placed in an uncovered aluminum DSC pan. The DSC chamber was purged with nitrogen for 5 min before polymerization. One minute after the beginning of the acquisition, the samples were irradiated for 2 min at 37 °C with a LED curing light (Bluephase, Ivoclar-Vivadent AG). The incident light intensity was 20 mW cm^−2^. Each experiment was repeated at least three times. The heat flux was monitored as a function of time using the DSC under isothermal conditions. The DBC was calculated as the quotient of the overall enthalpy evolved [Δ*H*_P_ (J g^−1^)] and the theoretical enthalpy obtained for 100% conversion of the mixtures [Δ*H*_0P_ (J g^−1^)] (Equation (1)).

(1) $$\text{DBC}=\Delta H_{\text{P}}/\Delta H_{\text{0P}}$$

Δ*H*_0P_ was calculated according to the following formula (Equation (2)):

(2) $$\Delta H_{\text{0p}}=\Sigma \Delta H_{\text{0i}}\cdot P_{\text{i}}/M_{\text{i}}$$

where Δ*H*_0i_ is the theoretical enthalpy of monomer *i* (*i* = methacrylate or methacrylamide, Δ*H*_0i_ = 54.8 kJ mol^−1^ [17]), *M*_i_ its molar mass and *P*_i_ the amount used in the formulation (wt%).

The rate of polymerization (*R*_p_) was calculated according to the following formula (Equation (3)):

(3) $$R_{\text{p}}=Q/\left(m\Delta H_{\text{0P}}\right)$$

where *Q* is the heat flow per second during the reaction and *m* the mass of the mixture in the sample.

### 2.6. SBS measurement

Freshly extracted bovine mandibular incisors were embedded in unsaturated polyester resin (ViscoVoss). Flat dentinal and enamel surfaces were prepared with 120-grit and 400-grit wet silicon carbide paper on the labial side of the embedded teeth. The adhesive was first rubbed on the prepared dentin or enamel surface with a microbrush for 20 s. The adhesive layer was strongly air dried and light cured for 10 s with a LED curing light (Bluephase G2, polywave LED with a spectrum from 385 to 515 nm and 2 maxima at 410 and 470 nm, Ivoclar Vivadent AG). A poly(ethylene) mold with a central 2.38-mm diameter circular hole was fixed on the surface. A composite (Tetric EvoCeram, Ivoclar Vivadent AG) was inserted in the mold and light-cured for 20 s. The samples were finally stored in water at 37 °C for 24 h before being tested. The SBS was measured using a universal testing machine (Zwick, Germany) at a crosshead speed of 0.8 mm min^−1^. 10 samples were tested for each adhesive.

## 3. Results and discussion

### 3.1. Syntheses

**Figure 2.**
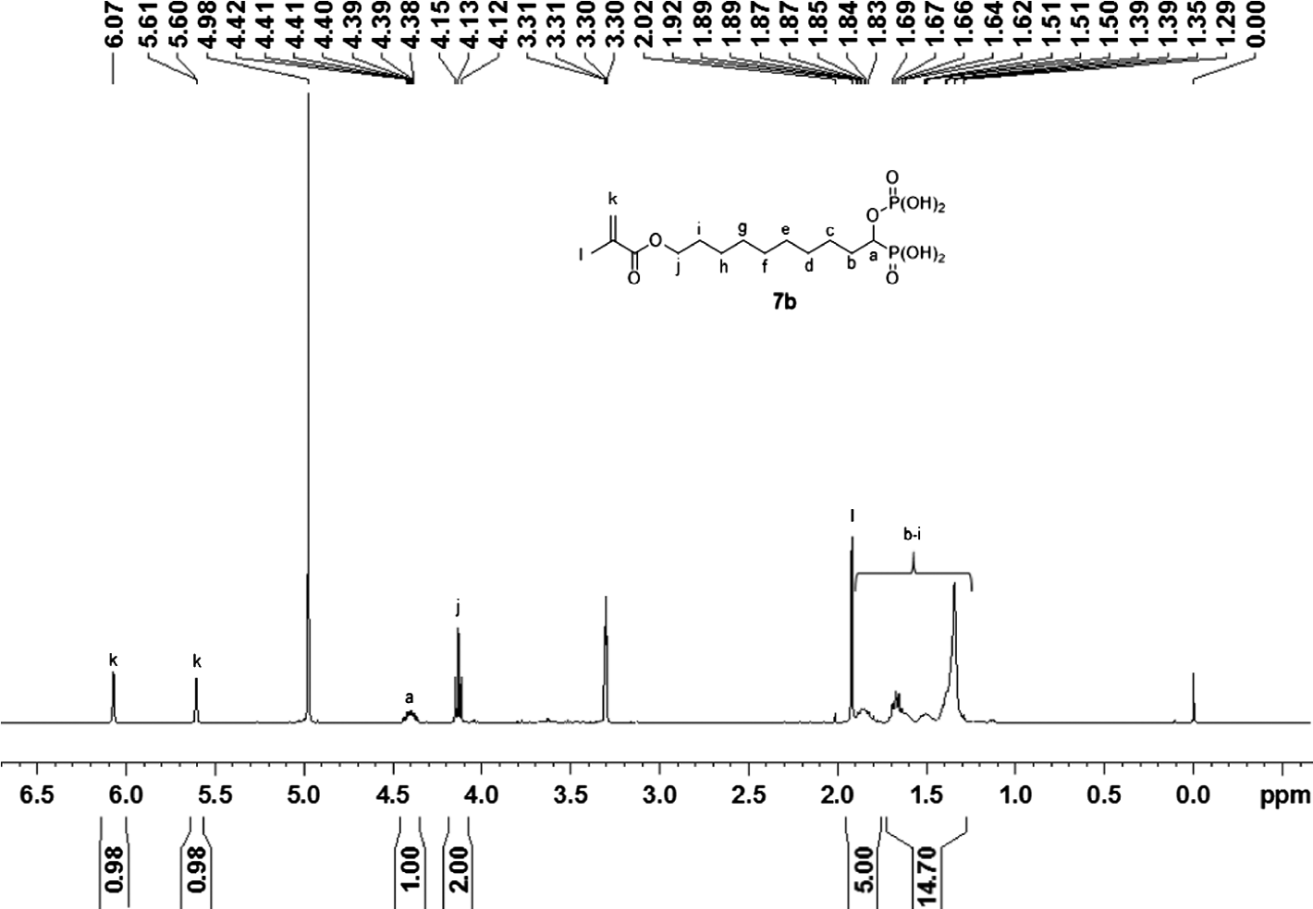
^1^H-NMR spectrum of monomer **7b.**

**Scheme 1.**
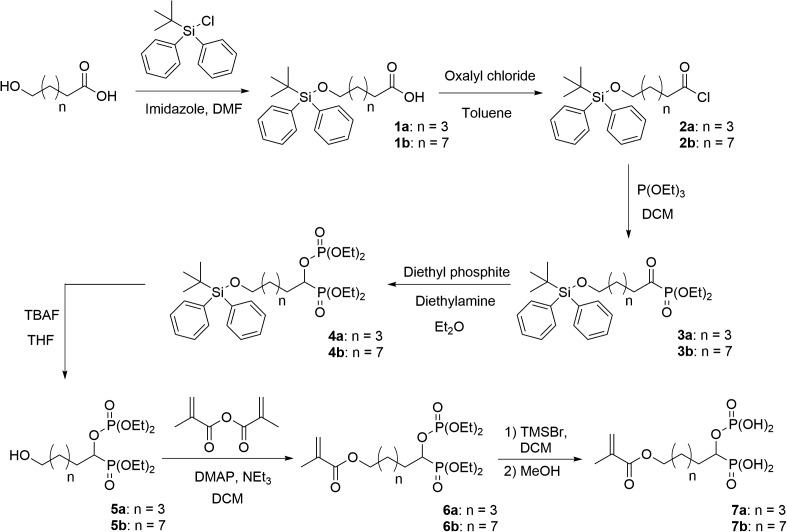
Synthesis of acidic monomers **7a** and **7b**.

**Scheme 2.**
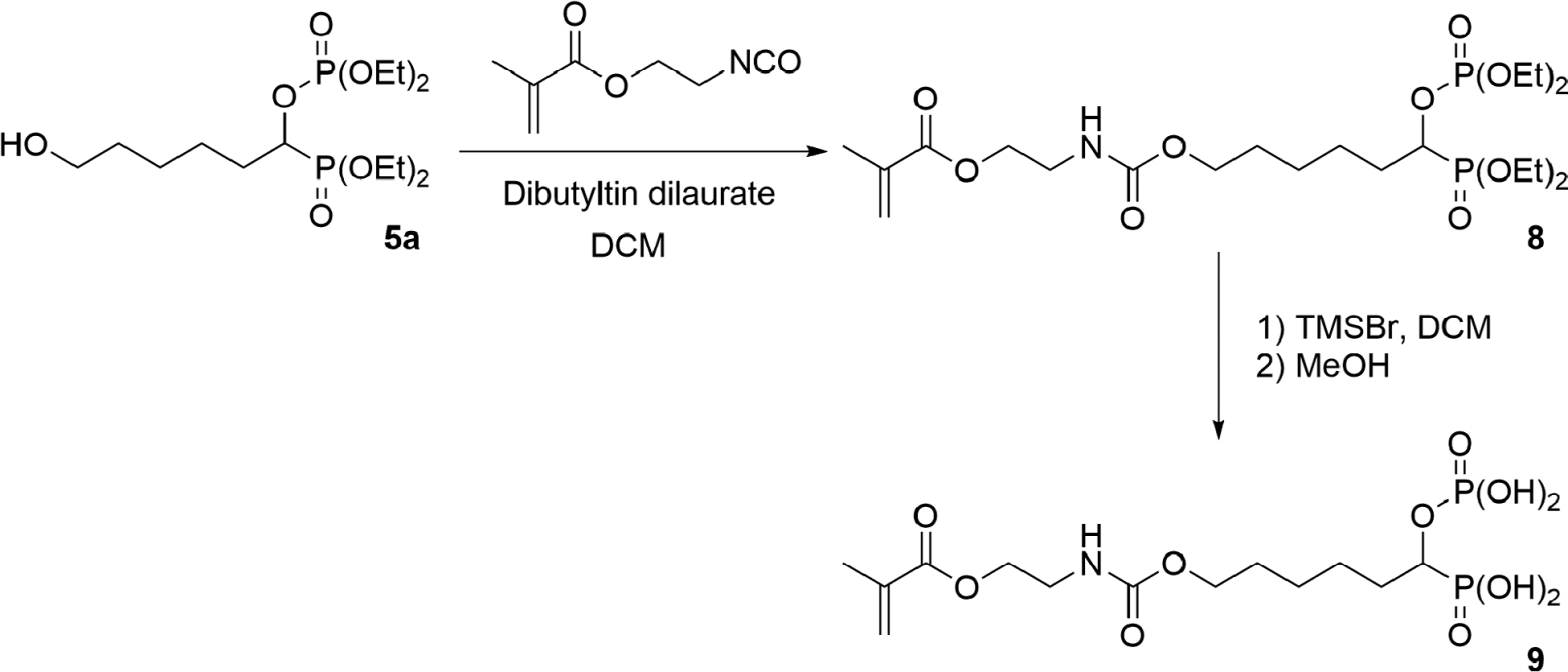
Synthesis of acidic monomer **9**.

**Scheme 3.**
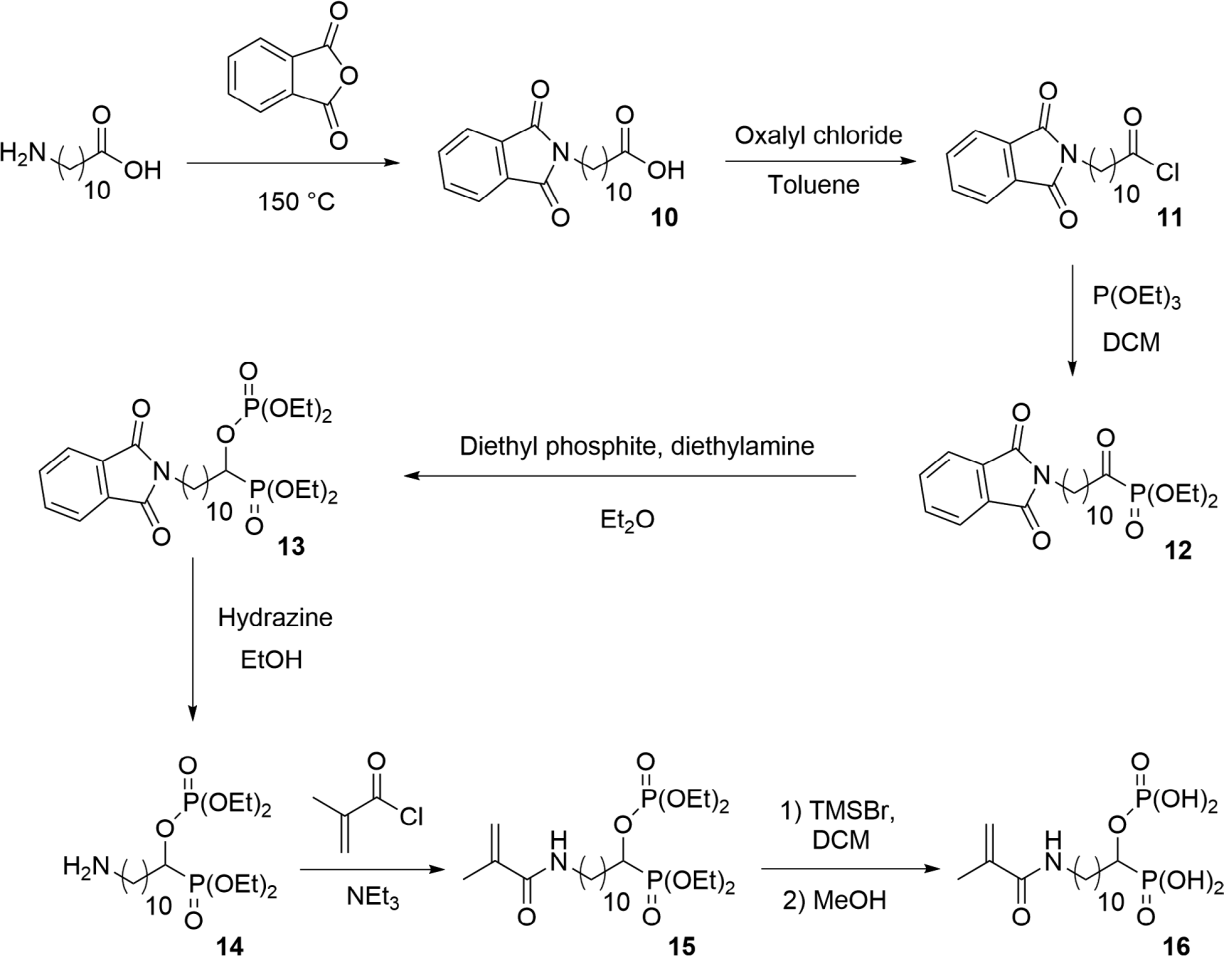
Synthesis of acidic monomer **16**.

Monomer **7a** was prepared, starting from 6-hydroxycaproic acid, in seven steps (Scheme 1). The alcohol group was first protected using *tert*-butyldiphenylchlorosilane and imidazole in DMF. The reaction between the resulting silyl ether and oxalyl chloride provided the corresponding acyl chloride **2a** in a quantitative yield. The addition of triethyl phosphite to the acyl chloride **2a** resulted in the formation of α-ketophosphonate **3a**. Gem-phosphonate-phosphate **4a** was subsequently prepared according to conditions reported by Nguyen et al. [15]. Hence, **4a** was synthesized by reacting **3a** with diethyl phosphite in the presence of a stoichiometric amount of diethylamine in diethyl ether. After purification by flash column chromatography, compound **4a** was isolated in 72% yield. Then, the *tert*-butyldiphenylsilyl protecting group was cleaved using TBAF in THF. The resulting alcohol **5a** was obtained in 86% yield. Methacrylate **6a** was synthesized by acylation of **5a** with methacrylic anhydride in the presence of triethylamine and of a catalytic amount of DMAP. Silylation of **6a** using TMSBr, followed by the methanolysis of the silyl ether, finally provided the desired acidic monomer **7a** in 95% yield. Monomer **7b** was prepared, from 10-hydroxydecanoic acid, according to a similar synthetic pathway (Scheme 1). It was isolated in a 47% global yield. Acidic monomer **9** was synthesized in two steps, starting from alcohol **5a** (Scheme 2). Methacrylate **8** was first prepared by reacting **5a** with 2-isocyanatoethyl methacrylate using dibutyltin dilaurate as a catalyst. It was obtained, after purification, in 93% yield. The dealkylation of the phosphonate and phosphate groups subsequently gave the desired monomer **9**. It is well-known that the use of (meth)acrylic monomers in SEAs present some drawbacks. Indeed, SEAs being acidic aqueous solutions, such monomers tend to hydrolyse upon storage. A low acidic monomer concentration or a storage in the refrigerator are required to prevent the deterioration of the adhesive. On the other hand, (N-alkyl)(meth)acrylamides are known to be significantly more stable than (meth)acrylates in aqueous acidic medium.[11,18–23] Therefore, such monomers have been incorporated in several commercially available SEAs. In this context, we took an interest in the seven-step synthesis of methacrylamide **16** (Scheme 3). The first step consisted in reacting 11-aminoundecanoic acid with phthalic anhydride at 150 °C. This reaction led to the formation of phtalimide **10**, which was obtained in 98% yield. The gem-phosphonate-phosphate **13** was then prepared in three steps, from carboxylic acid **10**, according to a similar synthetic pathway than the one previously described for compounds **4a** and **4b** (Scheme 1). The phtalimide group was successfully cleaved using hydrazine in EtOH. The corresponding amine **14** was isolated in 83% yield. A subsequent acylation using methacryloyl chloride, followed by deprotection of both dialkyl phosphonate and phosphate groups, finally led to the targeted acidic methacrylamide **16**. All new monomers were characterized by ^1^H-NMR, ^31^P-NMR and ^13^C-NMR spectroscopy. For example, the ^1^H-NMR spectrum of monomer **7b** is shown in Figure 2. It is characterized by nine methylene groups at 1.26–1.73 ppm (m, 14H), 1.75–1.96 ppm (m, 2H) and 4.13 ppm (t, 2H). The signal at 4.35–4.46 ppm corresponds to the proton of the CH bearing both acidic groups. The protons of the methacrylate group are identified by the presence of signals at about 1.92 (methyl group), 5.60 and 6.07 ppm.

### 3.2. Photopolymerization in bulk

**Figure 3.**
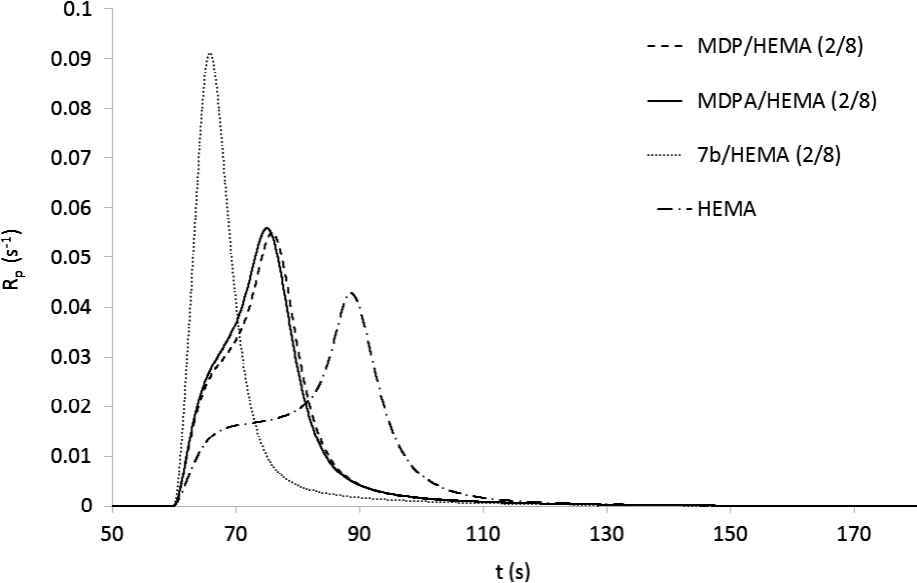
*R*_p_ vs. irradiation time for the polymerization of **7b**/HEMA, **MDP**/HEMA, **MDPA**/HEMA and HEMA.

In order to study the reactivity of acidic monomers **7a**, **7b**, **9** and **16**, their copolymerization with HEMA (acidic monomer/HEMA: 2/8, mol/mol) was investigated using photo-DSC. Each monomer mixture was polymerized under the same conditions (irradiation time: 2 min; light intensity: 20 mW cm^−2^). Ivocerin^®^ (0.5 mol%) was added as photoinitiator.[24] The copolymerization of MDPA and MDP with HEMA was also studied. For each mixture, the maximum rate of polymerization (*R*_pmax_), the time to reach the maximum rate of polymerization (*t*_Rpmax_) as well as the DBC were determined (Table 1). Figure 3 shows the rate of polymerization (*R*_p_) for **7b**/HEMA, **MDP**/HEMA, **MDPA**/HEMA and HEMA as a function of time. Results showed that the polymerizable α-phosphonooxy phosphonic acids **7a**, **7b**, **9** and **16** are significantly more reactive than MDP and MDPA. Indeed, higher *R*_pmax_ and lower *t*_Rpmax_ were obtained with these monomers. It can also be seen in Figure 3 that the addition of acidic monomers to HEMA results in a significant improvement of the reactivity. Contrary to MDP and MDPA, the new monomers **7a**, **7b**, **9** and **16** are bearing two acidic groups. It is well established that hydrogen bonding has a tremendous impact on the polymerization reactivity. Indeed, it is thought to facilitate the preorganization of the monomers, resulting in an increase of the propagation reaction rate constant (*k*_p_).[25–28] Hydrogen bonding also restricts mobility, leading to a decrease of the termination rate and consequently to an increase of the *R*_p_. The presence of two acidic groups strongly increases the ability of the monomer to form hydrogen bonds, which is probably responsible for the higher reactivity of monomers **7a**, **7b**, **9** and **16**. This property can also explain that the polymerization of the mixtures containing α-phosphonooxy phosphonic acids **7a**, **7b**, **9** and **16** led to lower DBCs (75.4% < DBCs < 80.5%) than for the mixtures based on **MDPA** (84.2%) and **MDP** (85.1%). Amongst the synthesized monomers, **9** was found to be the most reactive. This result can be explained by the presence of the additional urethane group, which can also participate in hydrogen bond interactions. No significant difference was observed between the reactivity of methacrylate **7b** and of the corresponding methacrylamide **16**. Results obtained with **7a** and **7b** also showed that the spacer length has almost no influence on the polymerization rate.

**Table 1.** *R*_pmax_, *t*_Rpmax_ and DBC measured for different HEMA comonomer mixtures.

Mixtures (mol/mol)	*t*_Rpmax_ (s)	*R*_pmax_ (s^−1^)	DBC (%)
**7a/**HEMA (8/2)	4.8	0.101 ± 0.001	80.5 ± 1.0
**7b/**HEMA (8/2)	4.8	0.091 ± 0.001	77.1 ± 1.0
**9/**HEMA (8/2)	3.8	0.105 ± 0.001	79.0 ± 0.1
**16/**HEMA (8/2)	5.8	0.085 ± 0.001	75.4 ± 1.0
**MDP/**HEMA (8/2)	15.4	0.055 ± 0.001	85.1 ± 2.0
**MDPA/**HEMA (8/2)	14.7	0.056 ± 0.001	84.2 ± 1.0

### 3.3. Adhesive properties

In order to evaluate the adhesive properties of monomers **7a**, **7b**, **9** and **16**, SEAs were formulated (Table 2). Each SEA contained an acidic monomer (15 wt%), crosslinking monomers (Bis-GMA and DEBAAP), photoinitiators (CQ/EMBO and Irgacure 819), solvents (water and *i*-PrOH) and additives (BHT as inhibitor). SEAs based on MDPA and MDP were also prepared. These adhesives were used to mediate a bond between a composite (Tetric EvoCeram^®^) and dental hard tissues (dentin and enamel). The SBS was subsequently measured (Table 3). SEAs containing the new polymerizable α-phosphonooxy phosphonic acids **7a**, **7b**, **9** and **16** provided significantly higher dentin and enamel SBS than the adhesives based on MDPA and MDP. The results confirm that the presence of two acidic groups has a considerable influence on the adhesive properties. Excellent chelating ability as well as improved etching properties of monomers **7a**, **7b**, **9** and **16** are probably responsible for the outstanding performances of SEAs 1–4. These adhesives led to similar dentin and enamel SBS. Consequently, neither the spacer length nor the nature of the polymerizable group nor the presence of a carbamate group had an influence on the SBS values. It should be emphasized that MDP is one of the most frequently used acidic monomer in commercial SEAs. α-Phosphonooxy phosphonic acids are therefore excellent candidates to improve the performance of current adhesives.

**Table 2.** Composition of six experimental SEAs.

Component	SEA 1 (wt%)	SEA 2 (wt%)	SEA 3 (wt%)	SEA 4 (wt%)	SEA 5 (wt%)	SEA 6 (wt%)
**7a**	15.00	–	–	–	–	–
**7b**	–	15.00	–	–	–	–
**9**	–	–	15.00	–	–	–
**16**	–	–	–	15.00	–	–
**MDP**	–	–	–	–	15.00	–
**MDPA**	–	–	–	–	–	15.00
DEBAAP	40.00	40.00	40.00	40.00	40.00	40.00
Bis-GMA	22.40	22.40	22.40	22.40	22.40	22.40
Water	15.00	15.00	15.00	15.00	15.00	15.00
*i*-PrOH	5.00	5.00	5.00	5.00	5.00	5.00
CQ	0.90	0.90	0.90	0.90	0.90	0.90
EMBO	0.42	0.42	0.42	0.42	0.42	0.42
Irgacure 819	1.25	1.25	1.25	1.25	1.25	1.25
BHT	0.03	0.03	0.03	0.03	0.03	0.03

**Table 3.** Results of dentin and enamel SBS tests.

SEA	Acidic monomer	Dentin SBS (MPa)	Enamel SBS (MPa)
1	**7a**	37.3 ± 5.1	29.1 ± 4.5
2	**7b**	36.5 ± 4.1	25.8 ± 2.8
3	**9**	38.5 ± 3.8	29.8 ± 3.0
4	**16**	33.8 ± 3.7	27.9 ± 2.9
5	**MDP**	27.7 ± 4.8	22.6 ± 4.0
6	**MDPA**	22.0 ± 3.3	16.4 ± 2.8

## 4. Conclusion

The polymerizable α-phosphonooxy phosphonic acids **7a**, **7b**, **9** and **16** were successfully synthesized in seven steps. The copolymerization of these monomers as well as of MDPA and MDP with HEMA was investigated. The addition of monomers **7a**, **7b**, **9** and **16** to HEMA led to an acceleration of the polymerization. Acidic monomers **7a**, **7b**, **9** and **16** were found to be significantly more reactive than MDP and MDPA. The strong ability of these monomers to form hydrogen bonds, which can be ascribed to the presence of two acidic groups, is thought to be responsible for their high reactivity. The adhesive properties of acidic monomers **7a**, **7b**, **9** and **16** were also evaluated. SEAs based on these α-phosphonooxy phosphonic acids led to significantly higher dentin and enamel SBS than adhesives containing the phosphonic acid MDPA or the dihydrogen phosphate MDP. Monomers **7a**, **7b**, **9** and **16** are therefore excellent candidates for the formulation of high performance adhesives.

## Disclosure statement

No potential conflict of interest was reported by the authors.
